# The Development and Integration of a Safety Officer Role to Facilitate Prevention of COVID-19 Virus Transmission in an Adult Inpatient Rehabilitation Setting Using Collaborative Change Leadership Methodology

**DOI:** 10.1177/21650799231186157

**Published:** 2023-07-20

**Authors:** Siobhan Donaghy, Jennifer Shaffer, Susan Schneider

**Affiliations:** 1 St. John’s Rehab Program, Sunnybrook Health Sciences Centre; 2 Department of Occupational Science and Occupational Therapy, Temerty Faculty of Medicine, University of Toronto; 3 Department of Physical Therapy, Temerty Faculty of Medicine, University of Toronto

**Keywords:** Collaborative Change Leadership, COVID-19 virus transmission, infection prevention and control, staff wellness

## Abstract

**Background:**

With the onset of the COVID-19 pandemic, a large urban academic hospital responded by creating the temporary role of a “Safety Officer (SO).” The key task of the SO role was to supervise staff donning and doffing personal protective equipment (PPE) and provide real-time feedback on their performance. The support for safe donning and doffing would contribute to staff well-being by reducing their fear of infection transmission.

**Methods:**

A Collaborative Change Leadership (CCL) approach was used to facilitate the development, implementation, and evaluation of the role. This included an iterative feedback process with clinicians and safety officers to continually refine the role.

**Findings:**

Feedback indicated value in the initiative as increasing staff confidence about preventing virus transmission, as well as their sense of safety at work. Areas for future improvement included additional communication strategies for interprofessional teams and external partners, as well as planning around logistics to better support the safety officers in performing this new, temporary role.

**Conclusions/Application to Practice:**

The Safety Officer role was able to help alleviate concerns regarding potential infection transmission and contribute positively to staff well-being.

## Background

The COVID-19 pandemic contributed to fears among many health professionals about their risk for occupationally acquired virus exposure and infection, including concerns about possibly exposing family members upon return home from work each day (Pollock et al., 2020). It is recognized that healthcare workers must have a safe and secure work environment to provide optimal care to patients and contribute to their overall sense of well-being (Belfroid et al., 2018). A recent review on workplace factors linked to mental health during the pandemic stressed the importance of building an organizational infrastructure to provide reassurance to staff that any risk of contamination would be proactively addressed and mitigated by the workplace (Giorgi et al., 2020). Specifically, the use of procedures to manage the risk of contracting a contagion in combination with the availability of personal protective equipment (PPE) was recognized as one tool to help alleviate the risk of team members experiencing mental health concerns (Giorgi et al., 2020).

As a result, clinical leaders at Sunnybrook Health Sciences Centre (Sunnybrook), a large, urban, multi-site academic hospital in Toronto, Canada, created a supplementary team-based infection control infrastructure through the development and temporary implementation of a safety officer (SO) role in the acute care programs of the hospital. The development and implementation of the role built on initial efforts by infection prevention and control (IPAC) practitioners to provide clinical teams with education regarding safe and effective donning and doffing of PPE, recognizing that such knowledge is crucial in preventing infection transmission to health care workers (Verma et al., 2020). Furthermore, evidence has shown it to be even more beneficial for one team member to watch their colleague don and doff PPE, so as to provide immediate feedback and alert them to any potential transmission (Díaz-Guio et al., 2020). The use of such a buddy system where there is mutual supervision has been recommended as a strategy to prevent potential errors in techniques which could make staff members more prone to infection transmission (Cheng et al., 2020). Furthermore, it has been suggested that this training take place in the same clinical setting where it is to be practiced, as it can facilitate realistic team-based communication, as well as allow for better retention of knowledge and sharing of skills among team members (Koh et al., 2020).

The St. John’s Rehab program of Sunnybrook provides adult specialty inpatient and outpatient rehabilitation including stroke, amputee, cardiac, musculoskeletal, trauma, and burn rehabilitation. It added services during the pandemic to provide inpatient rehabilitation to those experiencing challenges with daily functional performance, endurance, and ability to return to their communities following a severe COVID-19 infection with acute hospitalization (Wade, 2020). Initially, the SO role was implemented solely in the acute care programs of the hospital. With the addition of rehabilitation services for those recovering from severe COVID-19 infections, it was recognized that the SO role was needed to support the rehabilitation team. Clinical leaders in the rehabilitation program from the professions of nursing, occupational therapy, and physiotherapy responded to this identified need and embarked on a process to lead this initiative.

## Methods

A Collaborative Change Leadership (CCL) methodology was selected by the clinical leadership team to guide the time-sensitive creation of infrastructure to support staff and their well-being during this challenging and unprecedented time. This approach to change management equips leaders with meaningful ways to facilitate change through co-creation, by noticing, responding, and adapting to the needs that emerge within their teams (Centre for Advancing Collaborative Health care & Education [CACHE], 2022; MacPhee et al., 2013). It is an effective approach to use when faced with complex scenarios and is based on core concepts such as appreciative inquiry, generativity, and co-creation (CACHE, 2022; Scharmer, 2007). Focused on engaging stakeholders, it builds on strengths and allows for flexibility and adaptation as needs become apparent. In this initiative, the methodology facilitated an iterative change process, building from novel experience by leveraging the work of the hospital’s acute care partners, and co-creating an approach that would maximize its effectiveness in the rehabilitation setting (MacPhee et al., 2013; Quinn, 2004). This report describes the development and implementation of the temporary SO role, using the CCL methodology.

The CCL methodology is divided into three key domains: Creating, Implementing, and Evaluating. Each domain involves specific steps to ensure a collaborative approach.

### Domain 1: Creating

The initial step in this initiative involved the clinical leadership team first learning about the SO role description and education program that had been designed for the acute care sector of the hospital. A revised role description and educational program was then developed by the leadership team, by reviewing and adapting these materials to address the needs and context of the inpatient rehabilitation setting. Factors considered in this process included the types of staffing complements, patterns of patient activity, clinician workflows, and treatment environments encountered in this clinical setting.

### Domain 2: Implementing

This phase included two components. The first component focused initially on operational leaders identifying staff who could be redeployed to this temporary role and informing them of the change in their work duties and hours of work. It followed with more specific communication to SOs with instructions regarding work schedules, patient care unit locations they were being assigned to, and key team contacts. The second component included the delivery of educational sessions, in preparation for staff redeployment.

### Domain 3: Evaluating

This final domain focused on evaluating the role in the areas of staff competency and staff satisfaction. The evaluation consisted of both iterative and summative components.

Key to the success of this initiative was the iterative evaluation process which involved the leadership team meeting informally 2 to 3 times per week for check-ins with SOs during their shifts on the patient care unit. Key discussion questions were posed in an effort to learn about their experience and help build on their successes. Core CCL principles such as active listening, sensing, and adapting were key in this iterative process, as they helped to modify and clarify practical aspects of the role in real time, facilitating a positive experience for SOs and the teams they were supporting.

A formalized summative evaluation also took place approximately 3 months after completion of the implementation phase when admission volumes reduced, and it was determined that the temporary role could be discontinued. The summative evaluation addressed two areas of focus through the use of feedback surveys. The first area of focus addressed the SO experience, with survey question focused on their level of confidence with taking on the role, the length of training they received, and which aspects of training they felt were most effective. Using the CCL principle of appreciative inquiry, it also explored their perspectives on what worked well overall and what opportunities there may have been with the implementation of the role by collecting open-text comments (Table 2). The second area of focus included a survey to clinical teams, asking about staff well-being, as well as implications to their practice when having the support of the SOs as observers, real-time educators, and providers of feedback regarding PPE use (Table 3).

## Results

The CCL approach that was utilized to guide the overall process and development of this new and emerging role allowed the leadership team to pivot and respond to emerging needs, making continual adjustments and modifications to both the educational component and the tasks and logistics associated with the role.

### Domain 1: Creating

The SO role in the rehabilitation context was created and identified as one that would observe and assist with the safe donning and doffing of PPE when anyone entered or exited the room of a patient on droplet and contact precautions. SOs would need to ensure that the process was safe and effective, with staff members not skipping any steps or leaving it to routine practice and memory, by providing constructive, real-time feedback to those observed. Within the rehabilitation context, where patients often require access to specialized therapy equipment and resources that are shared between patients, SOs would additionally need to support staff members using such equipment to clean items properly, while also safely doffing their PPE after a therapy session.

Following the creation of the role description was the development of the staff training component. This was designed as an in-person session delivered by the interprofessional leadership team, representing the professions of nursing, occupational therapy, and physiotherapy. It initially mirrored what had been delivered in the acute care programs, as a full day session. After the first iteration, it became apparent that some components were more specifically applicable to the acute care context with welcome icebreakers and background information on the principles of interprofessional teamwork. In the rehab context, staff participating in the training were previously familiar with each other and well versed in the principles of interprofessional collaboration in their daily work. This allowed the leadership team to create an abbreviated 3-hour format, focusing primarily on the description of the SO role, the use of PPE, and specific rehab case studies.

### Domain 2: Implementing

The first component of the implementation phase included a review of clinical staff from a variety of health professional roles who were eligible and appropriate for redeployment to this temporary role on one designated patient care unit. Staff members were selected by operational leaders based on their availability for redeployment from other clinical units as a result of public health–related service reductions or their current need for modified work duties. Once staff were identified, they were informed by their manager of the initiative and their new temporary role. Reassignment to the SO role involved adjustment in work roles, locations, team members, and schedules, as many were required to suddenly move from daytime weekday hours to rotational, 12-hour shifts over a 7-day work week.

The second component of the implementation phase involved the delivery of the customized training package for the rehabilitation staff which consisted of five key areas of focus, including an overview of the SO role, a review of PPE and IPAC practices, hands-on practice, role plays, and rehabilitation-based case studies. Sessions ranged from 2 to 4 hours in length and were adjusted based on both the size and needs of each group, as well as iterative feedback after each previous session. Additional virtual sessions in assertiveness training were also incorporated, recognizing the potential power imbalances and challenges that might arise as newly trained SOs provided feedback to colleagues across professions, roles, and hierarchies within the organization (Okpala, 2021).

While exiting the room after a therapy session, SOs would help with the cleaning of therapy equipment while also ensuring the correct doffing of PPE by therapists. When patients needed to exit their isolation rooms to practice mobility and stair climbing in common areas, SOs also supported the cleaning of handrails and doorways after use.

### Domain 3: Evaluating

#### Formative Evaluation

During the formative evaluation process, members of the leadership team connected with SOs in real time to understand their experiences. They were asked what was going well, and what could be done differently. This data informed continual adjustments and clarity regarding daily processes and guidelines that were required. Updates were then communicated to the SO team and a digital communication hub for the SOs was created in response to the feedback received. This included an email distribution list and an electronic portal where resources such as frequently asked questions (FAQs) could be posted by the SOs and responded to by each other and the leadership team. SOs were encouraged to continually provide iterative, process-oriented feedback in this communication hub using the formative evaluation questions outlined in Table 1.

**Table 1.** Iterative Evaluation: Process-Oriented Feedback Questions for Safety Officers

Question	Description
1	What are you noticing?
2	What is working well?
3	What gaps (if any) are you seeing on a recurring basis?
4	What can we do to address or clarify recurring gaps for you and/or the team?

Informal feedback from the SOs in the early stages of implementation indicated that some staff members and external partners/visitors were questioning their role in providing feedback about the donning and doffing of PPE. There appeared to be a lack of understanding about the SO role in contributing to staff safety. As a result, communication tools were created by the leadership team to provide additional background rationale for the SO role, as well as dissemination of information to external partners who entered the building regularly, so that they would understand the role of the SOs and follow the guidance being offered. This included communications to nursing agency staff, correctional officers, and a limited number of patient family visitors who were permitted to enter the building on an exceptional basis.

While SOs were only in place on the designated COVID-19 unit, based on the success of this initiative, the leadership team soon learned that team members in other areas of the rehab program had heard of the benefits of the role and inquired if they could access it as well. Pivoting to address this emerging need, the clinical leadership team created a modified role with abbreviated training sessions of 90 minutes for team members on other units, equipping them with skills to perform peer safety checks on their units, which helped to instill confidence and ensure safety among peers. This also ensured that staff were informed of the purpose of the formal SO role and to welcome feedback regarding PPE use that they were being given when they encountered the SOs.

#### Summative Evaluation

The summative evaluation included two feedback surveys. One was directed to the SOs (Table 2), and another solicited input from the clinical teams that they supported (Table 3).

**Table 2.** Summative Evaluation: Experiential Feedback Questions to Safety Officers

Question	Description
1	If you were trained as a safety officer or informal safety champion, which training did you receive?
2	How effective did you find the following aspects of the training? • Overview of safety officer role • Review of infection prevention & control practices and PPE • Hands-on practice with donning and doffing observation and providing feedback • “Challenging” staff member role play • Case studies
3	What did you find most helpful about the training?
4	What opportunities for improvement can you suggest with respect to the training involved for this role?
5	What impact did the training have on the work of you and your team?
6	Were you an official safety officer in an assigned 12-hour shift role?
7	Which resources did you access? Select all that apply - Assertiveness training session - Email distribution list - Shared electronic resource folder - Q&A document
8	What worked well with respect to this role? How did the team benefit?
9	Were there any aspects of this role that could be enhanced? What could have been done differently?
10	Any further comments?

*Note.* PPE = personal protective equipment.

**Table 3.** Summative Evaluation: Feedback Questions to Clinical Teams

Question	Description
1	During your clinical work on the unit, did you have access to a safety officer to observe you donning and doffing your PPE?
2	How confident did you feel in ensuring optimal infection control precautions as you entered/exited a patient room?
3	What aspects of the safety officer role did you find most helpful? Check all that apply: • Being present 24 hours per day • Extra set of hands to help don/doff PPE, tying gowns, assisting with two-stage cleaning • Extra set of eyes to make sure all steps were followed in the correct order to ensure your safety • Immediate real-time feedback on donning and doffing PPE • Other:
4	How has your practice related to donning, doffing, and infection prevention & control changed as a result of having safety officers observe?
5	What impact did this role have on: • Your overall well-being (no impact, space, some impact, space, large impact) • Your ability to do your job effectively
6	Share any examples in regard to the impact the safety officer role had on your overall well-being or the ability to do your job effectively

*Note.* PPE = personal protective equipment.

For the first survey, out of 29 SOs trained, 41% responded. The results indicated that the training program was found to be effective, with more than 75% finding the hands-on practice to be the most valuable component.

One respondent from this survey indicated that as they progressed in their role and clinicians became more accustomed to their presence on the unit that “staff really appreciated that help and they repeatedly thanked us for doing that.” Another respondent also noted that the nurses and therapists appreciated their support as it “conserved PPE and eliminated the risk of staff members trying to come into and out of the room with PPE on or to rush to doff PPE where mistakes could be made just to retrieve a forgotten item.” In addition, we heard from another SO that it was beneficial that many of them “already had a level of established rapport” with team members from their regular clinical roles, and this continued as they took on this new role.

When asked what areas for improvement they could suggest, respondents offered many insights. Four SOs noted the negative impact on them personally as they shifted to longer, rotating day/night shift schedules. Those who were primarily working daytime weekday shifts of 7.5 hours in non-nursing clinical roles were moved into 12-hour shifts, with 7 days per week coverage. Feedback indicated that this sudden change had a significant impact on their work–life balance and their overall well-being, with one respondent noting that the transition to 12-hour shifts was “exhausting and cost us our health.” Some also described having a lack of access to safe places to rest and physically distance during their breaks on such an unfamiliarly long shift, which further contributed to their experience of exhaustion. Some respondents also noted that additional clarity with logistics, communication processes, and procedures would have been helpful. Having hospital-approved and standardized information posters with directions that supported or reinforced the instructions that SOs were offering verbally and through demonstration would have been helpful. Another example was having additional clarity regarding the incident reporting process, including timelines, roles, and responsibilities associated with completing one when there was as an observed breach of PPE use.

While they valued the “what if” role-playing scenarios that were offered as part of their training, respondents did note that it would be helpful to integrate scenarios that also included interactions with external partners. For example, scenarios related to interactions with patient transport and correctional staff who frequently entered the units would have better aligned with the experiences and challenges staff members faced daily.

For the second survey, 24 team members responded. The results indicated that 95% of respondents valued the additional real-time feedback from SOs and noted that it positively affected their practice and sense of safety at work. The number of respondents who described their level of confidence as “very good” in ensuring optimal infection control precautions as they entered/exited a patient room rose from 5 to 16 after they engaged with the SOs.

The clinical team survey results indicated that what they valued most from their experience with SOs was their overall presence and support. Ninety percent of respondents found value in having the extra set of hands with donning and doffing PPE and assisting with two-stage cleaning of shared equipment that was exiting the patient room. Also, 95% of respondents found value in the receipt of immediate real-time feedback. One clinician commented that the SOs “were very good at noticing if our patients were not wearing a mask when we were interacting with them, or at pointing out that I had forgotten to gown up when entering a new patient’s room.” Another respondent also noted that the process for donning and doffing had “become very automatic for me now. They [SOs] would say first step, second step third step . . . now that is what I hear in my head as I don and doff.”

Limitations included the sudden transition of staff members into these new roles with significant disruptions to their work–life balance. The need for additional role clarification and conflict resolution strategies was also identified, as each group learned about each other and adapted to working in a different way with new team members. Overall, the implementation of the SO role created a more positive and safe work environment during such unprecedented times.

## Discussion

The development and implementation of the temporary SO role has demonstrated to be an effective strategy in quickly addressing the emerging needs of staff during the COVID-19 pandemic. It appeared to provide valuable education and support, and, in turn, provided staff with an additional layer of safety and confidence in their work. The strength of the program was that it offered real-time feedback and education to staff regarding their donning and doffing practices, which staff members reported finding valuable. It also lead to overall greater knowledge and safety with implications for transmissions and outbreaks. The presence of the SOs also allowed for more fluid movement of consultant staff through multiple units of the hospital, without having to limit their contact only on a designated COVID or non-COVID unit, since the presence of the SOs was providing an extra layer of protection.

In future, this role would be utilized again under similar circumstances if the need was to arise. Building on the existing role description and experiences to date, it is recommended that SOs continue to be recruited among existing clinical staff, leveraging the benefits of an established level of rapport and trust with those who they would be supporting. Any future implementation of the role would need to ensure not only education for SOs but also for the teams that they are supporting, so that role clarity is well established as a foundational element for implementation. Training would also be expanded to include scenarios with external partners and would be supported with clear policy statements, processes, and communication tools, to ensure alignment across the organization.

The continued inclusion of assertiveness training as part of the training program for SOs was seen as an asset in addressing potentially challenging conversations regarding power imbalances that might present themselves. Finally, thoughtful consideration would be advised when planning work schedules and resources to better support rest breaks for staff transitioning to such a role in the future, being mindful of the impact on their work–life balance and overall well-being.

More broadly, consideration should be given to addressing team functioning and interprofessional collaboration when embedding and/or redeploying any staff member into an intact team, to ensure that appropriate supports and resources are available for all in the process.

The utilization of CCL principles was crucial in guiding the process and being able to respond and adapt as new issues and challenges emerged. Building on strengths and possibilities, and sensing when staff needed additional support, were key as the team embarked on this uncharted territory during the pandemic. Being able to build on existing relationships, facilitate co-creation, and make adjustments to the initiative in real time undoubtedly helped lead the team to success. If faced with another unexpected system level challenge with the same degree of uncertainty, the team would feel confident using the CCL approach again as an effective methodology for building an effective and resilient response.

**Applications to Professional Practice**

A Collaborative Change Leadership approach was used during the COVID-19 pandemic to implement a Safety Officer role to supervise donning and doffing of personal protective equipment at the rehabilitation site of a large urban teaching hospital. This role was able to help alleviate concerns regarding potential infection transmission and contribute positively to staff well-being. While this initiative successfully leveraged the CCL approach in a healthcare setting, there is potential for these principles to be applied to much broader occupational health contexts, as a tool to use when responding to significant workplace challenges requiring the need for effective and rapid change.
